# Phylogenetic relationships of sleeper gobies (Eleotridae: Gobiiformes: Gobioidei), with comments on the position of the miniature genus *Microphilypnus*

**DOI:** 10.1038/s41598-022-26555-7

**Published:** 2022-12-22

**Authors:** Isadola Eusébio Macate, Adam Bessa-Silva, Rodrigo Antunes Caires, Marcelo Vallinoto, Tommaso Giarrizzo, Arturo Angulo, Gorgonio Ruiz-Campos, Iracilda Sampaio, Aurycéia Guimarães-Costa

**Affiliations:** 1 Laboratório de Evolução, Instituto de Estudos Costeiros, Universidade Federal do Pará, campus de Bragança, Alameda Leandro Ribeiro, 68600-000 Bragança, Pará Brazil; 2 CIBIO-InBIO, Centro de Investigação em Biodiversidade e Recursos Genéticos, Universidade do Porto, Campus Agrário de Vairão, 4485-661 Vairão, Portugal; 3 Laboratório de Diversidade, Ecologia e Distribuição de Peixes, Instituto Oceanografico da Universidade de São Paulo, Praça do Oceanografico, Butantã, 05508-120, São Paulo, Brazil; 4 Laboratório de Biologia Pesqueira - Manejo de Recursos Aquáticos, Universidade Federal do Pará, Campus do Guamá, Av. Perimetral. 2651, Belém, Pará Brazil; 5 Museo de Zoología, Escuela de Biología, Universidad de Costa Rica, San Pedro de Montes de Oca, San José, Costa Rica; 6 Centro de Investigación en Biodiversidad y Ecología Tropical, Museo de Zoología, Universidad de Costa Rica, San Pedro de Montes de Oca, San José, 11501–2060 Costa Rica; 7 Facultad de Ciencias, Universidad Autónoma de Baja California, 22860 Ensenada, Baja California Mexico

**Keywords:** Evolution, Genetics, Ecology

## Abstract

*Microphilypnus* and *Leptophilypnion* are miniaturized genera within the family Eleotridae. The evolutionary relationships among these taxa are still poorly understood, and molecular analyses are restricted to mitochondrial genes, which have not been conclusive. We compiled both mitochondrial and nuclear genes to study the phylogenetic position of *Microphilypnus* and the evolutionary history and relationships of eleotrids. We propose that *Microphilypnus* and *Leptophilypnus* (a non-miniature genus) are not sister groups as suggested by previous studies, but rather separate lineages that arose in the early Eocene, with *Leptophilypnus* recovered as a sister group to the other analyzed eleotrids. In fact, *Microphilypnus* is currently associated with the Neotropical clade *Guavina/Dormitator/Gobiomorus*. We also identified a well-supported clade that indicated *Gobiomorus* and *Hemieleotris* as paraphyletic groups, besides a close relationship among *Calumia godeffroyi*, *Bunaka gyrinoides, Eleotris* and *Erotelis* species. This is the first comprehensive report about the evolutionary relationships in members of the family Eleotridae, including multiloci and multispecies approaches. Therefore, we provided new insights about the phylogenetic position of some taxa absent in previous studies, such as the miniature genus *Microphilypnus* and a recently described species of *Eleotris* from South America.

## Introduction

The family Eleotridae (Bonaparte, 1835), whose members are popularly known as “sleepers” and “gudgeons”, is the second most diverse fish family of the suborder Gobioidei, within the order Gobiiformes^1–4^. It comprises 139 species from 22 genera^5^ widespread in tropical and subtropical waters in the Neotropics, Africa, and the Indo-Pacific^4–6^. Most eleotrids inhabit brackish or freshwater environments, albeit a few species are truly marine^7^. Furthermore, some freshwater species have a marine larval stage returning to freshwater as juveniles^8^. They are carnivores that feed on crustaceans and other benthic invertebrates, small fishes, and insects, although the marine larval stages of some species feed on plankton^9,10^.

**Figure 1:**
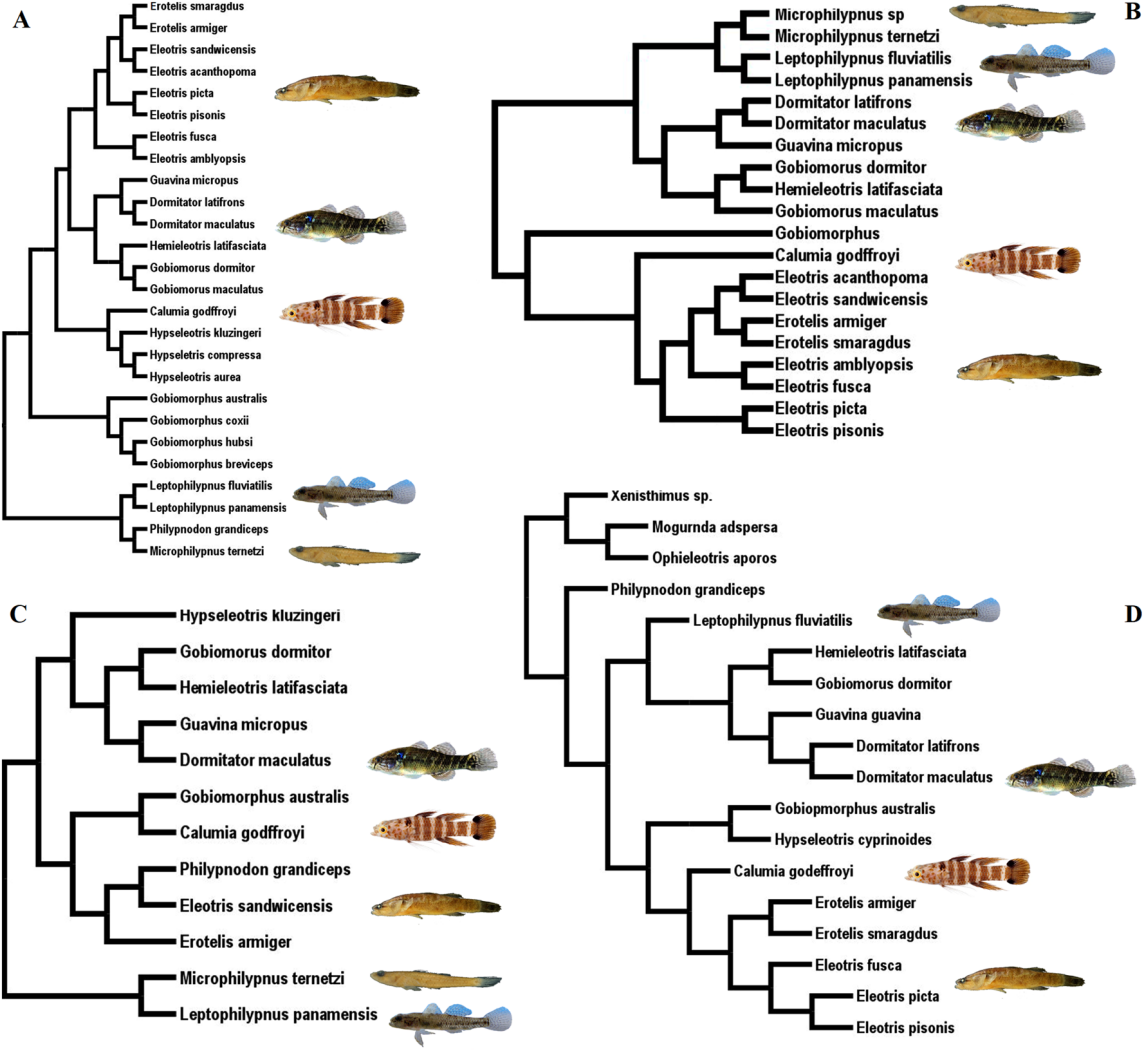
Previous phylogenetic hypotheses among eleotrids based on mitochondrial genes ((**A**) Thacker and Hardman (2005); (**B**)Thacker (2009); (**C**) Chakrabarty et al. (2012); (**D**) Thacker (2014)). Note the different positions of clades *Microphilypnus* (green), *Leptophilypnus* (blue), *Philypnodon* (red) and *Calumia* (yellow).

Molecular phylogenetic studies have provided significant insights into the evolutionary history of Eleotridae in the past decades, even though some phylogenetic relationships in this group still remain uncertain. For example, Thacker & Hardman^7^ suggested that the clade composed of *Microphilypnus*/*Philypnodon*/*Leptophilypnus* would be the sister-group of all other eleotrids, including the species from Oceania and the Indo-Pacific region (Fig. 1A). However, in general, this phylogeny was recovered with low support values for the relationships of the main groups of eleotrids. Nonetheless, Thacker^11^ noted that the clade *Microphilypnus*/*Leptophilypnus* appears to be the sister-group to the Neotropical clade (*Dormitator*/*Guavina*/*Gobiomorus*), while *Philypnodon* is more closely related to the genus *Gobiomorphus* from Oceania. In this case, recovering an almost fully resolved phylogeny with very good support values in general (Fig. 1B).

The phylogenetic position of the genus *Calumia* is also controversial. The authors^7^ proposed a clade formed by *Calumia* and *Hypseleotris* (Fig. 1A), while Thacker (2009)^11^ suggested a closer relationship between *Calumia* and *Eleotris* (Fig. 1B)*,* whose species have a circumtropical distribution. Subsequently, Chakrabarty et al.^12^ recovered *Calumia* as a sister group of the genus *Gobiomorphus*, native to New Zealand and Australia (Fig. 1C). However, this last relationship was also recovered with a low probability value.

Although these latter studies have shed light on general aspects of evolutionary relationships within the family Eleotridae, all of them were based solely on mitochondrial DNA (mtDNA). Outside of this mitochondrial scenario, Agorreta et al.^13^ used five molecular markers (two mitochondrial and three nuclear) for 222 species of gobioids. However, only 16 species of eleotrids were included in their phylogenies, and species of *Microphilypnus* and *Leptophilypnus* were not considered. Furthermore, the phylogenetic supermatrix proposed by McCraney et al.^14^ based on 23 loci, which corresponds to the most recent study and comprehensive on the phylogeny of Gobioidei, showed that the phylogenetic position of *Microphilypnus* and *Leptophilypnus* remains questionable.

Despite the recurrent use of mitochondrial sequences to solve long-standing phylogenetic problems over the past few decades, phylogenies based on single genes (considering that mtDNA behaves as a single genetic locus) provide limited phylogenetic signals and therefore single-locus inferences might be biased^15,16^. In this sense, combining nuclear (nuDNA) and mitochondrial DNA (mtDNA) genes can provide finer descriptions of phylogenetic and phylogeographic scenarios, being more efficient than studies based only on mtDNA markers^13,17–22^. Furthermore, it is now widely accepted that combining information from multiple loci using methods that account for stochastic processes during evolution improves the inferences about the historical diversification of organisms^23–25^.

### Miniaturization in eleotrids

Miniaturization is an evolutionary process that leads to reduced body size of lineages over time, being observed in several groups of fishes, amphibians, reptiles, and primates^26–28^. In general, miniaturization is accompanied by structural simplifications, novel structures, and increased variation^29^. In some cases, the truncated development eventually determines the appearance of distinct evolutionary novelties, including “bizarre” forms^30,31^.

The miniature taxa (traditionally defined as those species with a total length below 25 mm) are particularly diversified in ichthyofauna^32^. For example, only in the Neotropical region, nearly 210 miniature fish taxa have been reported encompassing the main orders: Characiformes, Siluriformes, Cyprinodontiformes, Perciformes, and Gobiiformes^33^. In the order Gobiiformes, two miniature genera of the family Eleotridae are recognized, namely: *Microphilypnus*^34–37^, and *Leptophilypnion*^38^.

Although the description of miniature forms and their adaptive relationships have been addressed since classical works by Haeckel^39^, current phylogenetic approaches might elucidate the tempo and mode of evolution in body size of species and/or lineages^40–42^. However, the few studies available based on molecular data involving the miniature genera *Microphilypnus* have generated contrasting evolutionary scenarios. The authors^7,11^ hypothesized a phylogenetic relationship between miniaturized species of the genus *Microphilypnus* and with non-miniaturized genus *Leptophilypnus* from coastal rivers in Central America. On the other hand, Thacker^43^ demonstrated that *Microphilypnus* is more closely related to another non-miniature genus (*Philypnodon*), endemic to freshwater ecosystems in Australia. Therefore, further investigations are required to provide a reliable phylogenetic reconstruction about the evolutionary transition from non-miniature to miniature groups, and thus, determine whether miniaturized genera represent a miniaturized clade or the result of independent evolutionary events of miniaturization.

Therefore, to refine the evolutionary relationships within Eleotridae, we generated a more comprehensive phylogeny of this family based on mitochondrial and nuclear genes, including taxa excluded from previous phylogenetic reports and comprising different biogeographic regions. Although molecular data from representatives of the miniature genus *Leptophilypnion* are still absent, our multi-locus phylogenetic analyses allow exploring the phylogenetic position of the miniaturized group *Microphilypnus*, which has often been neglected in former studies.

## Results

**Figure 2:**
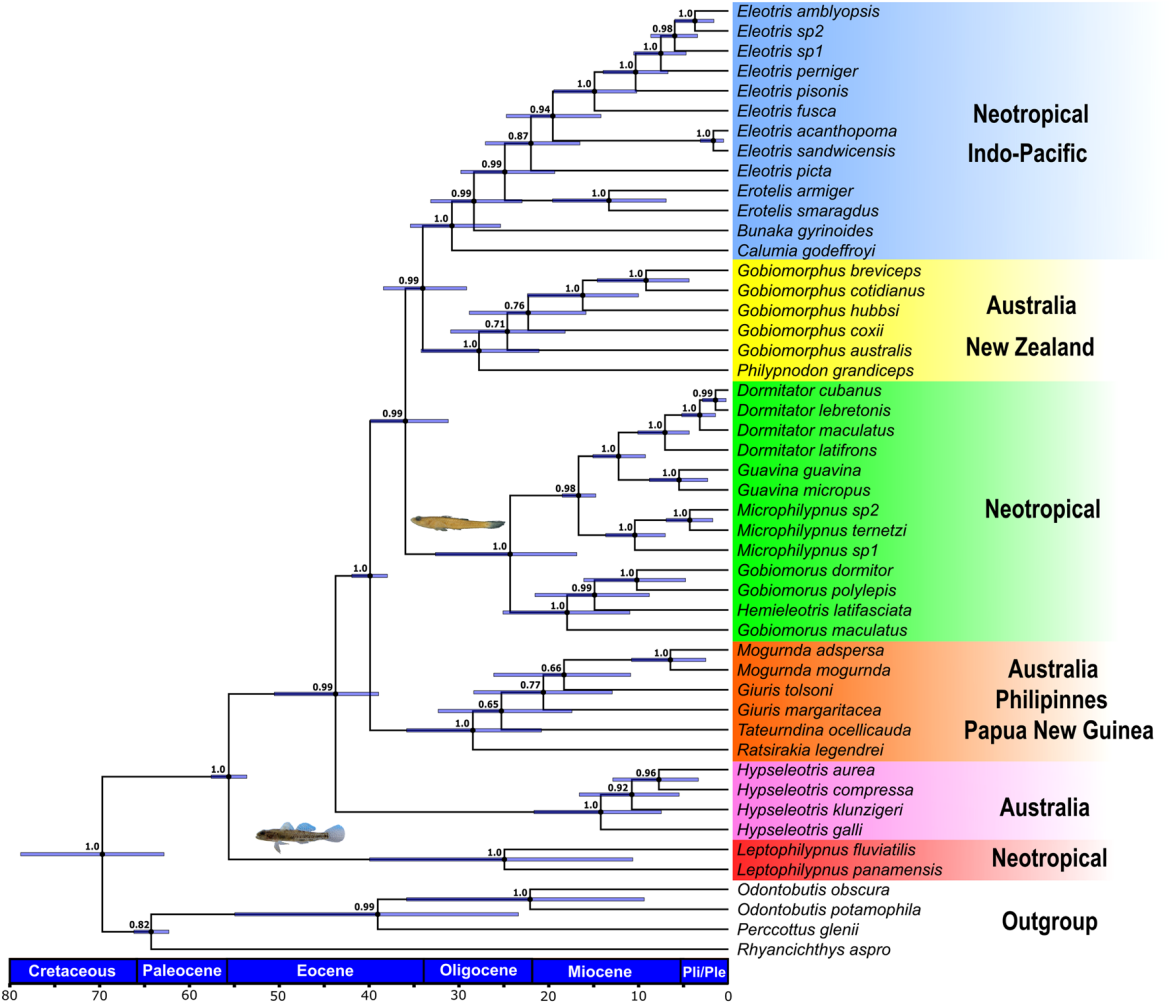
Combined mitochondrial and nuclear DNA species tree based on the algorithm implemented in *BEAST. Blue bars illustrate the 95% highest posterior density of node heights and support values of the posterior probability are displayed on each node. The clade in green shows the phylogenetic position of the miniature lineage of *Microphilypnus*, while *Leptophilypnus* (red branch) appears as sister group of other eleotrids.

The final dataset in molecular phylogenetic analyses consisted of 52 taxa, being four of them related to outgroups. The final concatenated alignment of mitochondrial and nuclear DNA sequences had a length of 3494 bp (16S, COI, ND2, Rhod, EGR1) and the phylogenetic reconstruction was based on the Bayesian coalescence approach (species tree on *BEAST) (Fig. 2). The two exons used had a total of 247 variable sites in 1240 bp (EGR1 = 118/811, and Rhod = 129/429). The dataset partitioning scheme and the nucleotide substitution models for multilocus phylogenetic analyses are shown in Supplementary Table S2.

The phylogenetic reconstruction confirmed the monophyly of the family Eleotridae and revealed six well-supported main clades (> 0.99 PP): (1) *Eleotris*/*Erotelis*/*Bunaka*/*Calumia*; (2) the Australian genus *Gobiomorphus*/*Philypnodon*; (3) the Neotropical genus *Dormitator*/*Guavina*/*Gobiomorus*/*Hemieleotris*/*Microphilypnus;* (4) the Australian genus *Giuris*/*Mogurnda*/*Ratsirakia*/*Tateurdina*; (5) genus *Hypseleotris*; and (6) genus *Leptophilypnus* (Fig. 2).

The first clade (in blue) includes *Eleotris* from Neotropical and Indo-Pacific regions. The relationships between *Bunaka gyrinoides* and *Calumia godeffroyi* and the species of the genus *Eleotris* and *Erotelis* were strongly supported (PP > 0.99). We found that *Eleotris* species from the western Atlantic (*E. amblyopsis*, *E. pisonis*, *E. perniger*, and the recently discovered lineages but not formerly described (*Eleotris* sp. 1 and *Eleotris* sp. 2) form a monophyletic group. From the biogeographic point of view, our phylogenetic analyses showed that the neotropical species are not monophyletic (blue, green and red clades). Instead, the clade from Australia and New Zealand (yellow clade) is a sister group of the *Eleotris* lineage (PP = 0.99). In this clade, *Gobiomorphus* (represented by species from Eastern Australia and New Zealand) was recovered as a sister group of *Philypnodon* (Eastern Australia). Also, the close relationship between *Guavina* and *Dormitator* (Neotropical region) was well supported (PP = 1.0), while *Dormitator latifrons* (from Eastern Pacific) has been recovered as a sister lineage in relation to *D. maculatus* (Western Atlantic), *D. cubanus* (Cuba) and *D. lebretonis* (Western Africa).

The STACEY and SpeciesDA analyses considering all species involved in this study using all the molecular data (both mtDNA and nDNA) recovered strong support (see Supplementary Fig. S1 online) for a species delimitation hypothesis in which all putative taxa within the species group were distinct. Besides, the multi-locus approach (STACEY), also recognized the same relationships demonstrated between the more internal clades, which reinforces the robustness of our results in the face of phylogenetic uncertainties evidenced in previous works.

The miniature fish of the genus *Microphilypnus* were placed in a phylogenetic framework with eleotrid species from different biogeographic regions, suggesting a close evolutionary relationship with high support values (PP > 0.98) between this group (represented by *M. ternetzi*, *Microphilypnus* sp. 1. and *Microrphilypnus* sp. 2) and the neotropical clade, comprising *Guavina*, *Gobiomorus* and *Hemieleotris* (non-miniature genera). The molecular phylogeny supports the paraphyletic nature of the genus *Gobiomorus*, since *Hemieleotris latifasciata* appears nested in the *G. dormitor*/*G. polylepis*/*G. maculatus* clade. Finally, our phylogenetic analyses recovered the dichotomy of the Neotropical freshwater species *Leptophilypnus fluviatilis* and *L. panamensis* as a sister group of all eleotrid species herein analyzed (PP = 1.0).

### Estimates of divergence time in Eleotridae

We estimated the origin of the clade Eleotridae back to Early Eocene (55.6 Ma, IC = 53.6–57.5 95% highest posterior density—HPD), which corresponds to the split between the ancestral lineage of *Leptophilypnus* and the remaining eleotrids, with the subsequent diversification events occurred during the transition from Oligocene to Pleistocene. The clade including the miniature *Microphilypnus* species and the neotropical lineages (*Dormitator*/*Guavina*) has diverged during the Miocene (mean estimated date 16.6 Ma, 95% posterior credibility interval = 14.7–18.5 Ma). The most recent divergence events have taken place in Pleistocene (1.4 Ma) between *Dormitator cubanus* (from Cuba) and *D. lebretonis* (from Eastern Central Atlantic), followed by the split between *Eleotris acanthopoma* (Southeastern Asia) and *E. sandwicensis* (Hawaiian Islands) (1.6 Ma) (Fig. 2). The divergence among the lineages from the intercontinental clade composed of *Philypnodon* (Eastern Australia) and *Gobiomorphus* (Eastern Australia and New Zealand) appeared to have occurred during the Oligocene (27.7 My 95% IC 21.0–34.2 My).

## Discussion

The present study consists of a robust phylogenetic reconstruction of the family Eleotridae based on multiple loci (mtDNA and nuDNA). Based on these results, we inferred phylogenetic hypotheses to shed light on the evolutionary history of freshwater and estuarine eleotrids, encompassing the evolutionary relationships among 48 species. Although multilocus analyses have been performed recently, some important questions remain unresolved. For example, McCraney et al.^14^ showed interesting results on the phylogenetic relationships of the large Gobiaria group but indicated some instability within the Eleotridae. The authors do not discuss the causes of this instability in detail, but probably they can be explained by complex evolutionary scenarios. Furthermore, in the broad phylogeny of McCraney et al.^14^ ancestral relationships within the Eleotridae family are uncertain. In this study, it is not possible to determine ancestral relationships between *Eleotris*/*Gobiomorphus*/*Dormitador*/*Guavina* (although this uncertainty extends to other clades within the family). Therefore, to resolve the remaining controversies from previous studies, in addition to using species not included in previous phylogenies, we focused specifically on the family Eleotridae, which simplifies the evolutionary reconstruction scenario. In this way, we believe that the results obtained here can help to clarify some of these points.

Miniaturization is a recurrent theme in evolutionary studies since this phenomenon involves processes related to the reduction of body size usually associated with remarkable changes in morphology, physiology, ecology, life history, behavior, and reproductive maturity of organisms^29^. From a genetic point of view, phylogenetic approaches can greatly contribute to unraveling the evolution of miniature species. For example, the phylogenetic position of the miniature genus *Paedocypris*, considered one of the smallest groups of vertebrates (standard length of 10–12 mm), was determined based on inferences from mitochondrial DNA (cytochrome b)^40^. These authors located *Paedocypris* as a sister group to the miniature species of the genus *Sundadanio*, both of which were found to be sister lineages and the other taxon within the family Cyprinidae. Later, Britz et al.^41^ provided a more consistent phylogenetic signal of this group based on six nuclear genes, where *Paedocypris* appears as a sister group to all cyprinids. Both reports indicated that the miniaturization processes have taken place independently.

In the case of Eleotridae, previous phylogenetic reconstructions based on mitochondrial genes corroborated *Microphilypnus* and the non-miniature genus *Leptophilypnus* as sister groups^7,11,12^, (Fig. 1). Here, we found strong support in the species tree that included the miniature species of *Microphilypnus* within the Neotropical clade *Dormitator*/*Guavina*/*Gobiomorus*/*Hemieleotris* (Fig. 2; Supplementary Fig. S1 online). However, as we did not include miniaturized *Leptophilypnion* representatives in our phylogeny, we cannot claim that *Leptophilypnion* is the sister group of *Microphilypnus*, and that miniaturization arose once in a clade, or twice independently throughout the evolution of the Eleotridae.

Apart from the fact that the phylogenetic position of the genera Microphylipnus and Leptophylipnus is still unclear (especially whether they are sister groups or not), no synapomorphy has yet been described for either taxon. Indeed, *Microphylipnus* exhibits a suite of morphological characters not found in Leptophilypnus and most other eleotrids, such as a reduction in pectoral fin rays (11–15 vs. 15 or more), a barely ossified lateral ethmoid (vs. ossified and conical in frontal view), and a no ossified adult scapula (vs. ossified). Actually, *Lepthophylipnus* shares some reducing features with *Microphilypnus*, such as the slender infraorbital region and the absence of a row of infraorbital papillae. However, it is likely that these features evolved repeatedly in both lineages as the result of independent miniaturization events.

It is noteworthy that miniaturization events are often reported in Gobiiformes, suggesting a trend in this group towards the reduction of body size and loss of some morphological traits associated with miniature forms. Besides, the parallel adaptive evolution to similar microhabitats eventually leads to homoplasy, thus hindering the establishment of reliable phylogenetic relationships based only on morphology. On the other hand, the emergence of miniature and phylogenetically divergent groups supports our hypothesis that the miniaturization processes in Eleotridae represent independent evolutionary pathways.

Unfortunately, the phylogenetic position of *Leptophilypnion,* a recently described genus of Neotropical miniature eleotrids^38^, remains obscure. According to morphological traits, *Leptophilypnion* would be more related to *Microphilypnus* than to *Leptophilypnus*, by sharing some features such as the reduction in the number of scales and pectoral fins. Nonetheless, *Leptophilypnion* is distinguished by the presence of elongated pelvic fin rays, five branchiostegal rays (vs. six in other eleotrids), and additional unusual characters in skeleton^38^. In this case, inferring the phylogenetic position of *Leptophilypnion* would be of great importance to elucidate the evolutionary relationships among these species within Eleotridae, specially to clarify a question that remains uncertain, namely, whether miniaturization events within eleotrids arose independently or consisted of ancestral traits. However, after numerous collections at the sites where the holotypes were found (Negro and Tapajós Rivers—Brazil, according to Roberts (2013)^38^, we were unable to find the specimens of the two valid *Leptophilypnion* species. Alternatively, we tried through partnerships with ichthyological collections and other research groups to obtain these species, but unfortunately, we were not successful. Therefore, more information is needed to clarify the evolutionary relationships between species in these groups.

*Eleotris* corresponds to the only genus of Eleotridae that is widely distributed in different biogeographic areas, from the Neotropics, Africa, Indo-Pacific to Oceania. Differently to the previous reports^7–11^, which found a close relationship between *Eleotris amblyopsis* and *Eleotris fusca*, the new taxa included in our phylogenetic analyses indicated that *Eleotris amblyopsis* is the sister species of *Eleotris* sp.2, a newly discovered lineage in Nothern coast of Brazil^6^. We also recovered *B. gyrinoides* and *C. godeffroyi* within the clade *Eleotris*/*Erotelis*. Both species are distributed in the Indo-Pacific region and have a disjunct range when compared to the Neotropical genera *Eleotris* and *Erotelis*. Based on the presence of 10 + 15 vertebrae and pterygiophores of first dorsal fin beginning on the third interneural space, in a series of 1, 2, 2, and one element respectively (combination 3(1221)), the genera *Eleotris*, *Erotelis* and *Calumia* had been referred to the group “*Eleotris*”^44^, which also includes freshwater and estuarine species of *Belobranchus* from Indo-Pacific*.* However, to fathom the fascinating evolutionary history of these genera of eleotrids, robust approaches are needed to determine evolutionary diversification and its relationships to past environmental conditions. For example, the role of dispersal and/or vicariant events in the distribution and phylogeographic structure of these species should be carefully investigated.

Our data revealed a close phylogenetic relationship between the genera *Guavina* and *Dormitator*, which has also been reported in previous studies^7,11,12^. Morphological evidence also supports these results since both genera share one unambiguous synapomorphy first two hemal spines curved, arched (see Birdsong^44^^)^. On the other hand, *D. latifrons* and *D. maculatus* were not recovered as sister species, thus differing from previous reports^8,11,45^. The inclusion of new species in this study, i.e. *D. lebretonis* and *D. cubanus* resulted in a close relationship between species from the Atlantic Ocean, following the same trend observed in the diversification of *Eleotris*, in which the Atlantic species (*D. maculatus*, *D. cubanus* and *D. lebretonis*) form a monophyletic group. Therefore, our results corroborate the previous inference by Galván-Quesada^46^.

According to the present molecular analyses, *Gobiomorus* is paraphyletic in relation to *H. latifasciata*, as also indicated by Thacker^11^. *Gobiomorus dormitor* (Western Atlantic) and *G. polylepis* (Eastern Pacific) are also sister species, representing a didactic example of geminate species that diverged after the formation of the Isthmus of Panama. The origin of Eleotridae dates to Eocene (55.6 My), but their ancestral area remains unknown because sister groups to this family were not included in this study. Our phylogenetic analysis successfully recovered the species from Eastern Pacific and Western Atlantic (*Erotelis armiger*/*E. smaragdus*; *Guavina guavina*/*G. micropus*; *Gobiomorus polylepis*/*G. dormitor*; *Leptophilypnus fluviatilis* / *L. panamensis*) as sister groups, similarly to the results obtained by Thacke^45^. The time-calibrated phylogeny showed that the lineages diverged before the formation of the Isthmus of Panama 3.1 Mya^47^. However, the most recent speciation events (1.4 Mya) occurred between *D. cubanus*, endemic to Cuba, and *D. lebretonis* from “Western-Central Atlantic.

Regarding the miniaturized clade *Microphilypnus*, the estimate of divergence time of the clade *Dormitator*/*Guavina* was approximately 16.6 Mya. Lovejoy et al.^48^ considered *M. ternetzi, Dormitator,* and *Eleotris* as marine-derived taxa*,* representing an endemic remnant of ancient radiations to Neotropical freshwater habitats. This result is in accordance with the origin of freshwater lineages from marine ancestors driven by sea level fluctuations in South America coast during Cretaceous-Eocene^48–50^. Many clades, such as *Plagioscion* (Sciaenidae), *Jurengraulis* and *Anchovia* (Engraulidae), and *Pseudotylosurus* (Belonidae), have probably evolved from marine lineages by the connections formed between the Caribbean Sea and the Upper Amazon basin during this period via Los Llanos basin and Pebas Lake in Venezuela^51–55^.

Similarly, the paraphyletic status of *Gobiomorphus* in relation to *Hemieleotris* herein observed agrees with other reports. However, we suggest caution before the synonymization of these lineages since unambiguous synapomorphies for the clade *Hemieleotris* and *G. polylepis*/*G. dormitor* have not been described. In this context, subsequent radiations throughout the South American basins determined a profusion of morphologically and ecologically distinct species not seen in marine habitats^56^. Exploring this biogeographic background is highly recommended to understand the miniaturization process in Eleotridae. In fact, smaller body sizes in freshwater taxa when compared to marine forms have been widely reported, with explanations ranging from the advantages of reduced size in offering greater maneuverability in structured environments^57^ to the reduction of energetic demands in size-constrained or complex microhabitats^31^.

In summary, these data provide the most complete hypothesis for Eleotridae phylogeny to date, because it includes representatives from several biogeographical regions. Our results were based on evolutionary information from mitochondrial and nuclear genes, and then, revealed a novel phylogenetic relationship from previous studies based only on mtDNA. The miniaturization does not seem to be a frequent event in Eleotridae, because the miniature taxa evolved in at least two genera (*Microphilypnus* and *Leptophilypnion*). As a result, we propose that miniaturization is an evolutionary process in the genus *Microphilypnus* with a strongly supported sister group relationship between *Microphilypnus* and the neotropical genus *Guavina*, *Dormitator* and *Gobiomorus*. As the position of *Leptophilypnion* was not established in the phylogeny, we cannot affirm the close relationship between the miniature taxa. Thus, more extensive taxonomic and geographical sampling and analysis based on multi loci may reveal whether this event is exclusively part of a clade. The non-miniature genus *Leptophilypnus* was often considered to be a sister group of the *Microphilypnus*, however, our results are consistent with the hypothesis that both lineages evolved independently.

## Material and methods

### Taxon sampling

A total of 48 samples were included in the phylogenetic analyses, being 22 of them collected in the wild and 26 obtained from NCBI GenBank (Table S1). The dataset consisted of 22 species of Eleotridae found exclusively in the Neotropical region, including members of all currently recognized genera, except *Leptophilypnion*^38^, which has a relatively recent description with no genetic data available in public databases.

To explore the phylogenetic relationships hypothesized in the previous studies^7,11,46^, we also included DNA sequences of species from other biogeographical regions, such as Indo-Pacific, Australia, New Zealand, New Guinea, Madagascar, and Africa. Four species of Gobiiformes (*Perccottus glenii*, *Odontobutis potamophila*, *Odontobutis obscura* and *Rhyacichthys aspro*) were used as outgroup. All newly acquired sequences were deposited in GenBank (accession numbers in Supplementary Material 1).

### Ethical statement

The samples analyzed in the present study were obtained in accordance with the requirements of Brazilian environmental legislation, being approved by the federal Chico Mendes Institute for Biodiversity Conservation (ICMBio), through license number 38047–3. The individuals were euthanized (according to the Brazilian legislation, law 11,974, being authorized by the Ethics Committee of ICMBio followed by Federal University of Pará) using an anesthetic application (5% Lidocaine) over the skin to minimize animal suffering, as recommendations of the American Society of Ichthyologists and Herpetologists.

### DNA extraction, PCR, and genomic sequencing

Total genomic DNA was extracted from muscle tissue using the Wizard Genomic DNA Purification kit (Promega Corporation, Madison, WI, USA). The Polymerase Chain Reaction (PCR) was carried out to obtain 2254 base pairs (bp) of three mitochondrial markers: ~ 565 bp of 16S rRNA gene (16S) 16S-L1987 5′-GCCTCGCCTGTTTACCAAAAAC-3′ and 16S-H2609 5′-CCGGTCTGAACTCAGATCACGT-3′^58^; ~ 697 bp of cytochrome c oxidase I (COI) GOBYL5490—5′-ATGGGGCTACAATCCACCGCTT-3′ and GOBYH7127 5′-ACYTCTGGGTGACCAAAGAATC-3′^7^; ~ 992 bp of NADH dehydrogenase subunit 2 (ND2) GOBYL4035 5′-CCCATACCCCAAACATGTCGGTTA-3′ and GOBYH5513 5′-GAGTAGGCTAGGATTTTWCGAAGYTG-3′^7^ and 800 pb of two single-copy exons: ~ 429 bp of rhodopsin gene (RHOD) RH28F: 5'-TACGTGCCTATGTCCAAYGC-3' and RH1039R 5'-TGCTTGTTCATGCAGATGTAGA-3'; and ~ 371 bp of early growth response 1 (EGR1) E1290F 5'-TMTCTTACACAGGCCGYTTCAC-3' and E11126R 5-CTTTYTCTGCTTTCTTGTCCTTCT-3′^59,60^. The amplification reactions were performed in a final volume of 25 µL, containing 4 µl of the dNTP (1.25 mM), 2.5 µl of 10 × buffer solution, 1 µl of MgCl_2_ (25 Mm), 0.25 µl of each primer (200 ng/µl), 1 µl of template DNA (100 ng/µl), 1 µl of Taq DNA polymerase (5 U/µl) and 15 μL of ultrapure water.

For the mtDNA markers, the PCRs conditions were as follows: initial denaturation at 94 °C for 4 min, followed by 35 cycles of 40 s at 94 °C, 40 s of annealing, 72 °C for 3 min, and a final extension of 5 min at 72 °C. The amplification conditions for nuDNA included an initial denaturation at 94 °C for 5 min, followed by 40 cycles of 94 °C for 40 s for denaturation, 30 s at 50 °C for annealing (16S, ND2, and COI), and 40 s at 52 °C for annealing (RHOD and EGR1), and 72 °C for 90 s for extension, plus a final extension of 7 min at 72 °C. The efficiency of amplification via PCR was checked in a 2% agarose gel. Amplified products were purified with PEG (polyethylene glycol) and sequencing reactions were performed with the BigDye reagent kit. The purified samples were then sequenced by the Sanger method^61^ using an ABC 3500xL automatic sequencer (Applied Biosystems).

### Phylogenetic analyses

The sequences were aligned automatically using MUSCLE^62^, as implemented in GENEIOUS 9.0.5 (https://www.geneious.com). The phylogenetic analyses were performed based on concatenated mitochondrial and nuclear partitions but applying separate priors. The aligned sequences of multiple loci were concatenated using SequenceMatrix 1.7.8^63^. The best-fit evolutionary model was selected in PartitionFinder 2^64^ for each gene and for each codon position in the case of protein-coding genes. The best-fit partitioning schemes and models are shown in Supplementary material 2.

### Estimates of divergence times and species tree

The analysis of TMRCA (Time of the Most Recent Common Ancestral) as well as a species tree (*BEAST)^65^ were implemented in *BEAST 2.5.2^66^. We used the five genes according to the optimal partitioning strategy as indicated by PartitionFinder 2 (Table S2). The simulations were carried out assuming an uncorrelated lognormal relaxed molecular clock, and the Yule speciation process as a prior^67^. The BEAST analysis comprised two independent runs, using 10 million generations, sampled every 5000 generations. The first 10% of all samples were removed as burn-in, and Tracer 1.7.1^68^ was used to check the effective sample sizes (ESS) assuming optimal parameters (> 200). The maximum credibility tree was generated in TreeAnnotator v1.6.1^69^. The resulting phylogenetic trees were visualized in Figtree 1.4.3^70^. The TMRCA was estimated based on the recovered ages in the study developed by Betancur-R^71^. These authors calibrated points from the fossil record using a subset of 202 taxa, 18 genes, and 59 calibration points. Based on this study, we used the origin of the family Eleotridae (mean age of 55.47 Ma) as a calibration point.

To validate the multilocus phylogenetic taxonomy, we performed an analysis in STACEY 1.2.5^72^ implemented in Beast 2.6.2. We conducted STACEY analysis using the previously described StarBEAST2 dataset, with all taxa and partitions conserved in both analyses (Supplementary Table S2). Final phylogenetic relationships were estimated in four independent runs for the whole data set. Each run consisted of 50 million iterations and parameter estimates sampling every 10,000 generations, discarding the first 10% as burn-in. STACEY log files were examined in Tracer v.1.7.1^67^ to assess whether the runs have reached the stationary phase and converged on model parameters (ESS > 400). Support of topologies was evaluated in STACEY by constructing a tree of maximum reliability in TreeAnnotator after the rejection of half of all estimated trees. Species delineation (based on the trees evaluated in STACEY) was carried out using a Java-application speciesDA (http://www.indriid.com/software.html), using simcutoff 1 and collapse height 0.0003.

## Supplementary Information

Supplementary Information.

## Data Availability

The datasets generated and analyzed in the current study are available in GenBank (GenBank accession numbers are shown in supplementary material).
