# Supplementary material for: Phylogenetic relationships of sleeper gobies (Eleotridae: Gobiiformes: Gobioidei), with comments on the position of the miniature genus Microphilypnus
Source: Sci Rep. 2022 Dec 22;12:22162. doi: 10.1038/s41598-022-26555-7 (PMC9780216; doi:10.1038/s41598-022-26555-7)
Supplement: Supplementary file 1 — Supplementary Information. [file 41598_2022_26555_MOESM1_ESM.docx]

**Phylogenetic relationships of sleeper gobies (Eleotridae: Gobiiformes: Gobioidei), with comments on the position of the miniature genus** *Microphilypnus*

Isadola Eusébio Macate, Adam Bessa-Silva, Rodrigo Caires, Marcelo Vallinoto, Tommaso Giarrizzo, Arturo Angulo, Gorgonio Ruiz-Campos, Iracilda Sampaio and Aurycéia Guimarães-Costa

**Supplementary Material**

**Table S1**. Composition, biogeographic region, and number of access to the GenBank of the Eleotridae species analyzed in this study. The sequences generated in this study are highlighted in bold.

| **Species** | **Biogeographic Region** | **16S** | **COI** | **ND2** | **Rhodopsin** | **EGR1** |
| --- | --- | --- | --- | --- | --- | --- |
| *Eleotris* sp1 | Amazonas River, Santana - Brazil | **KX095203** | **KR029121** | - | **KU204046** | **KU204052** |
| *Eleotris* sp2 | Cruçá estuary, Curuçá - Brazil | **KX095202** | **KR029130** | - | **KU204045** | **KU204051** |
| *Eleotris pisonis* | Southwest Atlantic | **KX095204** | **KU727863** | - | **KU204047** | **KU204053** |
| *Eleotris perniger* | Western Atlantic | **KX095207** | **KU727873** | - | **KU204049** | **KU204055** |
| *Eleotris picta* | Eastern Pacific | **KX095205** | **KR029135** | AY722349 | **KU204048** | **KU204054** |
| *Eleotris amblyopsis* | Western Atlantic | - | AY722154 | AY722354 | - | - |
| *Eleotris sandwicensis* | Hawaiian Islands | - | AF391333 | AF391477 | - | - |
| *Eleotris acanthopoma* | Southeast Asia and Northeast Oceania | AP004455 | AP004455 | AP004455 | - | KC826880 |
| *Erotelis armiger* | Eastern Tropical Pacific | - | - | AY722366 | - | - |
| *Erotelis smaragdus* | Western Atlantic | **KF415354** | AF391355 | AF391499 | **ON068595** | - |
| *Eleotris fusca* | Indo-Pacific | **KX095206** | **KU727889** | AY722369 | **KX095230** | - |
| *Guavina micropus* | Eastern Central Pacific | KF415390 | AY722131 | AY722331 | - | - |
| *Guavina guavina* | Western Atlantic | **KX095208** | **KU727830** | - | **KX095231** | - |
| *Dormitator maculatus* | Western Atlantic | **KX095209** | **KU727824** | AY722344 | **KX095232** | **KX095225** |
| *Dormitator cubanus* | Cuba | - | **ON067454** | - | KU765113 | - |
| *Dormitator latifrons* | Eastern Pacific | **KX095211** | **KX095219** | AY722343 | **KX095233** | **KX095226** |
| *Dormitator lebretonis* | Eastern Central Atlantic | - | - |  | KU765129 | - |
| *Gobiomorus maculatus* | Eastern Pacific | **KX095212** | **KU727883** | AY722375 | **KX095235** | - |
| *Gobiomorus polylepis* | Eastern Central Pacific | **KX095213** | **KX095221** |  | **KX095236** | **KX095228** |
| *Gobiomorus dormitor* | Western Atlantic | **KX095214** | **KU727886** | AY722345 | **KF235528** | - |
| *Hemieleotris latifasciata* | Eastern Central Pacific | **KX095215** | **KX095223** | AY722370 | **KX095234** | **KX095227** |
| *Microphilypnus* sp1 | Xingu River – Brazil | **ON072518** | **ON067455** | - | - | ON068597 |
| *Microphilypnus* sp2 | Amazonas River - Brazil | - | EU381040 | EU381019 |  | - |
| *Microphilypnus ternetzi* | Guyana | - | AY722181 | AY722378 | - | - |
| *Leptophilypnus panamensis* | Eastern Central Pacific | - | AY722127 | AY722326 | - | - |
| *Leptophilypnus fluviatilis* | Western Central Atlantic | - | AY722128 | AY722328 | - | - |
| *Gobiomorphus australis* | Eastern Australia | **ON072519** | **HM006958** | AY722348 | - | - |
| *Gobiomorphus coxii* | Eastern Australia | **ON072520** | **KJ669472** | AY722351 | **ON068596** | - |
| *Gobiomorphus hubbsi* | New Zealand | - | AY722156 | AY722358 | - | - |
| *Gobiomorphus breviceps* | New Zealand | - | AY722152 | AY722352 | - | - |
| *Gobiomorphus cotidianus* | New Zealand | KF415376 |  | AB560911 | - | - |
| *Philypnodon grandiceps* | Eastern Australia | **KF415442** | KJ669581 | AF391530 | - | - |
| *Hypseleotris aurea* | Eastern Australia | - | AF391392 | AF391536 | - | - |
| *Hypseleotris compressa* | Australia | KF415398 | KJ669477 | AF391510 | - | - |
| *Hypseleotris klunzingeri* | Eastern Australia | NC_043852 | NC_043852 | AF391537 | - | - |
| *Hypseleotris galii* | Eastern Australia | - | KJ669481 | AF514377 | - | - |
| *Giuris tolsoni* | Philippines | - | JN021219 | AF514368 | - | - |
| *Giuris margaritacea* | Indo-West Pacific | - | KU692515 | AY722361 | **KF235527** | - |
| *Mogurnda adspersa* | Eastern Australia | NC_024058 | AF391367 | AF391511 | - | - |
| *Mogurnda mogurnda* | North-western Australia | - | HM006969 | AY722341 | - | - |
| *Tateurndina ocellicauda* | Papua New Guinea | KF415480 | AY722175 | AY722372 | - | - |
| *Ratsirakia legendrei* | Eastern Madagascar | DQ532950 | AY722162 | AY722362 | - | - |
| *Calumia godeffroyi* | Indo Pacific | KX095201 | MF415023 | AY722325 | KF235529 | - |
| *Bunaka gyrinoides* | Indo-West Pacific | KX095200 | MN069307 | - | KX095229 | - |
| *Rhyancichthys aspro* | OutGroup | AP004454 | AP004454 | - | KF235519 | - |
| *Perccottus glenii* | OutGroup | KF415440 | KC292213 | AY722368 | KX224242 | - |
| *Odontobutis potamophila* | OutGroup | MK408452 | KF305680 | AY722371 | JQ937991 | - |
| *Odontobutis obscura* | OutGroup | AB095531 | JX679046 | AB560920 | - | - |

**Table S2**. Result from Partitionfinder for best partitions of mitochondrial and nuclear genes, and each codon position.

| **Mitochondrial genes** | **Best Model Select** |
| --- | --- |
| COI_1st codon position | SYM+G |
| COI_2nd codon position | F81+G |
| COI_3nd codon position | GTR+I+G |
| ND2_1st codon position | GTR+I+G |
| ND2_2st codon position | GTR+I+G |
| ND2_3st codon position | GTR+I+G |
| **Nuclear genes** |  |
| Rhodopsin_1st codon position | K80+I+G |
| Rhodopsin_2st codon position | JC+I+G |
| Rhodopsin_3st codon position | GTR+G |
| EGR1_1st codon position | GTR+I+G |
| EGR1_2st codon position | HKY+I |
| EGR1_3st codon position | HKY+G |


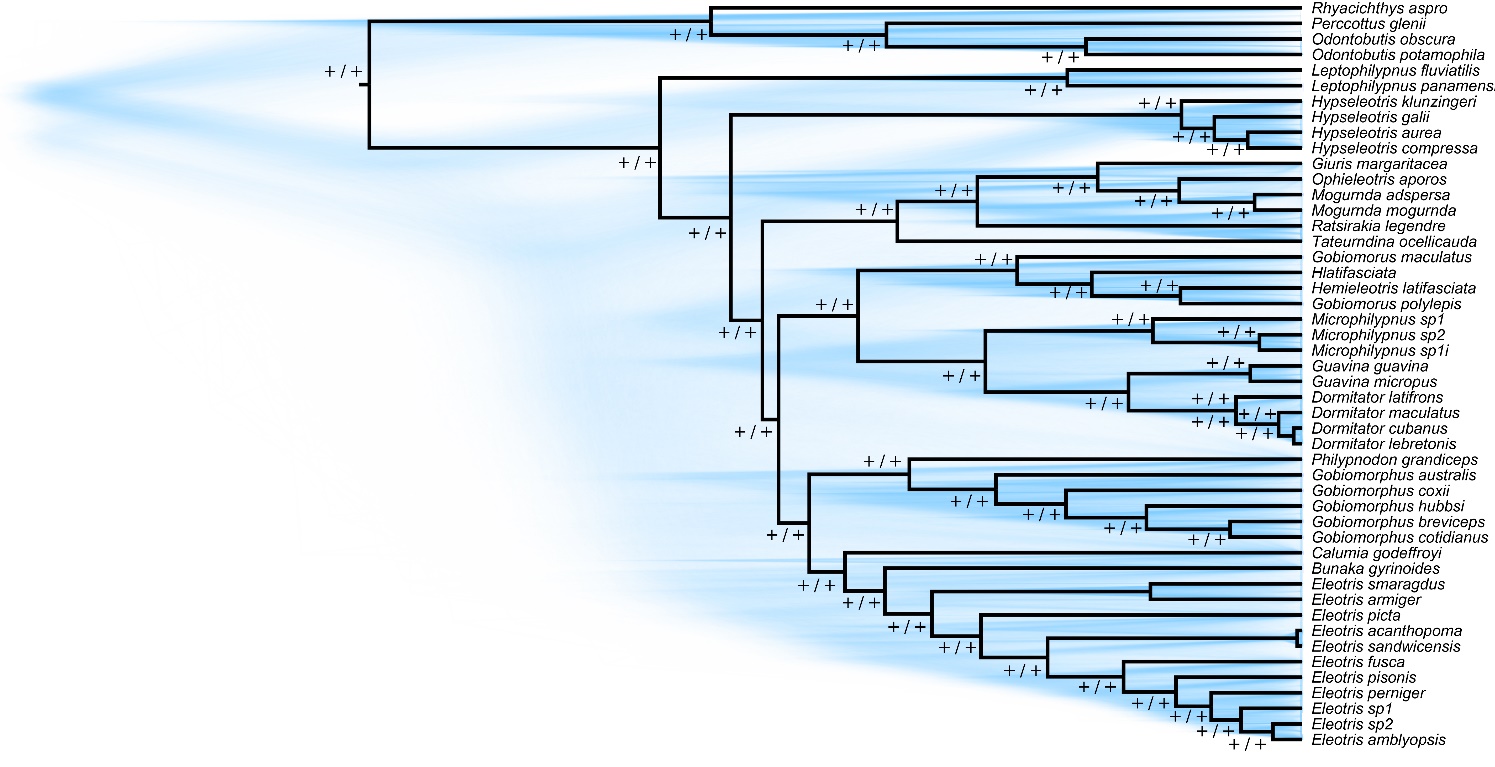


Figure S1. DensiTree representation of STACEY species-delimitation analysis using the subsampled StarBEAST2 dataset for all species used in this study, including the outgroups . Plotted onto well-supported nodes across the tree are + or - indicating whether the node is delimited in either one or both parallel STACEY analyses with SpeciesDA using either all genes (mtDNA and nDNA) or just the nDNA data.
